# Ru/GCN Nanocomposite as an Efficient Catalyst for Hydrogen Generation from Sodium Hypophosphite

**DOI:** 10.3390/nano14141187

**Published:** 2024-07-12

**Authors:** Ron Shirman, Sourav Chakraborty, Yoel Sasson

**Affiliations:** Casali Center of Applied Chemistry, Institute of Chemistry, The Hebrew University of Jerusalem, Jerusalem 9190401, Israel; ron.shirman@mail.huji.ac.il (R.S.); sourav.chakraborty@mail.huji.ac.il (S.C.)

**Keywords:** ruthenium nanoparticles, hydrogen evolution, hypophosphite decomposition, graphitic carbon nitride, catalysis

## Abstract

Sodium hypophosphite is a promising green source for generating clean elemental hydrogen without pollutants. This study presents the development of an efficient heterogeneous catalyst, Ru/g-C_3_N_4_ (Ru/GCN), for hydrogen generation from sodium hypophosphite. The Ru/GCN catalyst demonstrates excellent activity under mild reaction conditions and maintains its effectiveness over multiple cycles without significant loss of activity. This easily separable and recyclable heterogeneous catalyst is straightforward to operate, non-toxic, eco-friendly, and provides a cost-effective alternative to the extensive use of expensive noble metals, which have limited industrial applications. The Ru/GCN catalyst was characterized using various material characterization and spectral methods, including powder X-ray diffraction (PXRD), Fourier transform infrared (FTIR), thermogravimetric analysis (TGA), transmission electron microscopy (TEM), scanning electron microscopy with energy dispersive X-ray spectroscopy (SEM-EDS), and X-ray photoelectron spectroscopy (XPS). Hypophosphite, combined with the catalytically active and recyclable Ru/GCN catalyst, forms a system with high potential for industrial-scale hydrogen production, suggesting promising avenues for further research and application.

## 1. Introduction

The introduction of greenhouse gases (GHGs) into the atmosphere due to the continuous burning of fossil fuels poses a serious threat to the global environment and contributes to climate change [1,2]. For the long-term treatment of climate change, along with the reduction of dependence on oil imports, future energy sources must meet the requirements of being carbon-free and renewable [3]. In pursuit of this goal, several studies have focused on providing a clean and reliable alternative to traditional fossil fuels, leading to the development of hydrogen technology [4]. Hydrogen production under mild conditions is crucial for the economic viability and environmental sustainability of hydrogen energy systems. Combining hydrogen economics and the demand for clean hydrogen production highlights the importance of developing efficient and cost-effective catalytic systems. According to Guan et al., the cost of producing green hydrogen is a major barrier, with high electricity consumption accounting for most of the expenses. The study suggests that green hydrogen could potentially reach a future cost of around USD 1 per kilogram, provided that advancements in power-generation technologies and catalyst efficiency are achieved [5].

However, utilizing hydrogen gas as a fuel presents challenges such as its unavailability in nature, its explosive nature, and the need for inexpensive production methods [6,7]. Fortunately, a wide variety of processes are available for H_2_ production, utilizing hydrogen donor molecules such as formic acid [8,9,10], formate salts [11,12,13], water [14,15,16], liquid organic hydrogen carriers (LOHCs) [17,18], and others [19,20,21,22]. In this context, the development and application of nanomaterials play a crucial role in advancing hydrogen production technologies [23].

Hydrogen production from such carriers necessitates the utilization of catalysts. Extensive research has been conducted on heterogeneous carbon-based catalytic systems, specifically catalytic metals supported on carbon-based materials such as coal [24], graphene oxide [25], and other materials [26,27,28,29,30]. Graphitic carbon nitride (GCN) serves as a widely adopted support material for metallic nanoparticles owing to its exceptional properties, including outstanding chemical and thermal stability, an electron-rich structure, and a high nitrogen content that facilitates superior binding of metal particles [31,32,33,34]. The unique mesoscopic properties of GCN make it an ideal candidate for applications in nanotechnology and catalysis. Over the past few decades, the major catalytic methods employed for this reaction have included Pd, Pt, Rh, Ru, Ni, Cu, Co, and occasionally bimetallic catalysts [35,36]. Of these, Pd has emerged as the catalyst of choice for hydrogen generation reactions. However, a notable drawback associated with Pd is its elevated cost, and, in some instances, catalyst deactivation may occur due to metal oxidation and alterations in the particle size distribution of the metal on the support [37]. While perovskite LaCoO3 electrocatalysts have shown high oxygen reduction reaction (ORR) activity and stability in Al–air batteries, GCN-based catalysts exhibit higher efficiency in hydrogen evolution reactions (HERs) due to the synergistic effects between the GCN support and metal nanoparticles, enhancing catalytic activity and stability [38,39].

**Figure 1:**
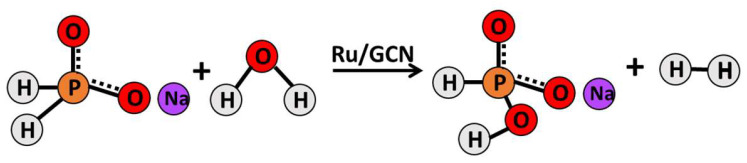
Representative flow diagram of the Ru/GCN catalyzed dehydrogenation of sodium hypophosphite.

In addition to the previously mentioned hydrogen carriers, hypophosphite has emerged as a promising source for hydrogen generation due to its commercial availability at a low cost and its reputation for safety and environmental friendliness [40,41]. What makes hypophosphite particularly appealing is its ability to release clean hydrogen without producing any byproducts, and it boasts a stoichiometric potential for hydrogen production that is twice as high as that of other sources such as potassium formate. In our prior research, we advocated for the use of sodium hypophosphite as a source of elemental hydrogen while conducting comparative studies of various metals supported on GCN as catalysts for the process [42]. Our findings favored palladium as the preferred metal; however, considering the necessity to transition to an industrial scale, the high cost of palladium poses a significant obstacle. The next best-performing metals were ruthenium and nickel, with the latter being somewhat unstable and prone to redox reactions, which can directly impact the entire catalytic system. Hence, in this current study, we propose the utilization of Ru/GCN as a catalyst for hydrogen production from hypophosphite (Figure 1). This study not only emphasizes the catalytic efficiency of Ru/GCN but also showcases the significance of nanomaterials in enhancing the performance and stability of catalytic systems. We undertook the characterization of the catalyst using various spectroscopic methods, optimized the process conditions to achieve maximum hydrogen yield, and presented a proposed catalytic mechanism for the process.

## 2. Experimental Procedure

### 2.1. Synthesis of Graphitic Carbon Nitride (GCN)

A total of 10 g of 99.99% urea (Sigma-Aldrich Ltd., Rehovot, Israel) was placed in an alumina crucible and heated at a rate of 10 °C/min up to 550 °C, and this temperature was maintained for 3 h. After the calcination process, the sample was cooled to room temperature at a rate of 10 °C/min. The final product obtained weighed 0.45 g.

### 2.2. Synthesis of Ru/GCN

In a round-bottomed flask, 500 mg of GCN was mixed with 150 mL of deionized water and stirred for 4 h at room temperature to exfoliate the GCN. An aqueous solution of ruthenium (III) chloride was then prepared and added to the mixture, followed by the addition of an aqueous solution of the reducing agent, sodium borohydride. The resulting mixture was stirred at 30 °C for 24 h. The produced catalyst was then isolated by centrifugation and washed thoroughly multiple times using a mixture of deionized water and acetone. Finally, the solid catalyst (Ru/GCN) was dried in an oven at 70 °C for 16 h.

### 2.3. Hypophosphite Decomposition Reaction for Release of H_2_

The mixture was stirred at 990 rpm and maintained at different temperatures for 30 min while connected to a reflux system with flowing ice water. This setup was linked to a flow-meter system that monitored the amount of hydrogen emitted. Upon completion of the reaction, the catalyst was centrifuged, thoroughly washed with deionized water and acetone, dried overnight at 70 °C, and then recycled for another round of use.

To assess the catalytic performance of the tested systems, we analyzed their conversion percentages. The conversion percentage quantifies the proportion of reactants that have been converted into products during the reaction, thereby indicating the catalytic efficiency. This percentage is calculated using the formula provided in Equation (1), where ${n(r)}_{0}$ denotes the initial moles of the reactant added to the system and ${n(r)}_{end}$ represents the moles of the reactant remaining after the reaction concludes.

(1) $$\%Conversion=\frac{{n(r)}_{0}-{n(r)}_{end}}{{n(r)}_{0}}\times 100\%$$

## 3. Results and Discussion

### 3.1. Material Characterization

**Figure 2:**
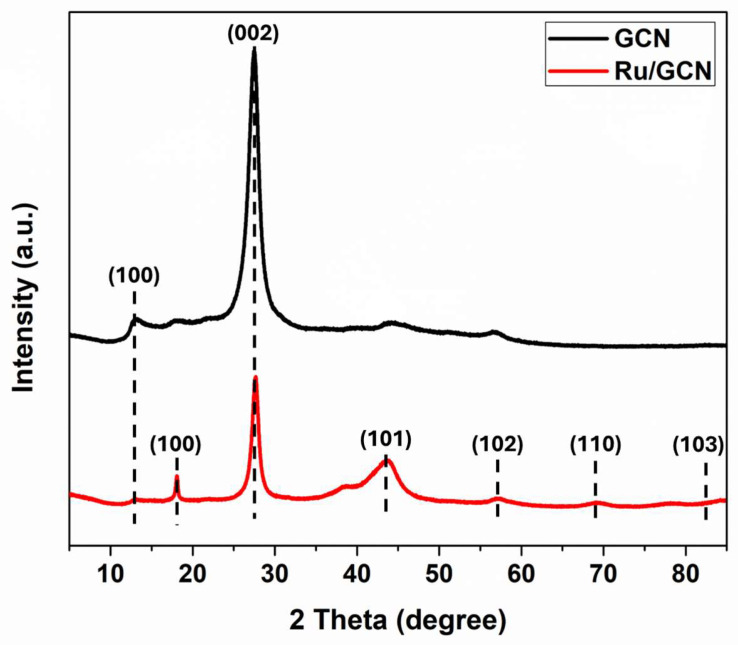
PXRD patterns of GCN and Ru/GCN.

The fabricated materials, GCN and Ru/GCN, were characterized by various physicochemical and microscopic techniques. To study the chemical composition, which provides details regarding the phase purity of the prepared material, we investigated the powder X-ray diffraction (PXRD) patterns, as shown in Figure 2. The PXRD pattern of GCN shows two characteristic peaks at 12.87° and 27.39°. The diffraction peak at 12.87° corresponds to the (100) diffraction plane, which is related to the in-planar repeated tri-*s*-triazine units of GCN. The strong peak (002) around 27.39° is attributed to the interplanar d-spacing and π–π stacking of the conjugated aromatic system. For Ru/GCN, it can be seen that the two typical peaks of the GCN conjugated aromatic layers are present. In addition, the main characteristic peak located at ~43.51° can be indexed to the (101) planes of metallic Ru nanoparticles. Additional peaks of Ru are located at ~57.71°, 69.19°, and 83.89°, and correspond to the (102), (110), and (103) planes. All four of these peaks indicate the hexagonal structure of ruthenium (JCPDS card no. 89-3942). In addition, the unassigned peak suggests that a bioorganic phase crystallizes on the surface of the nanoparticles. The crystallite size of Ru/GCN was observed to be 1.81 nm according to the Scherrer equation.

**Figure 3:**
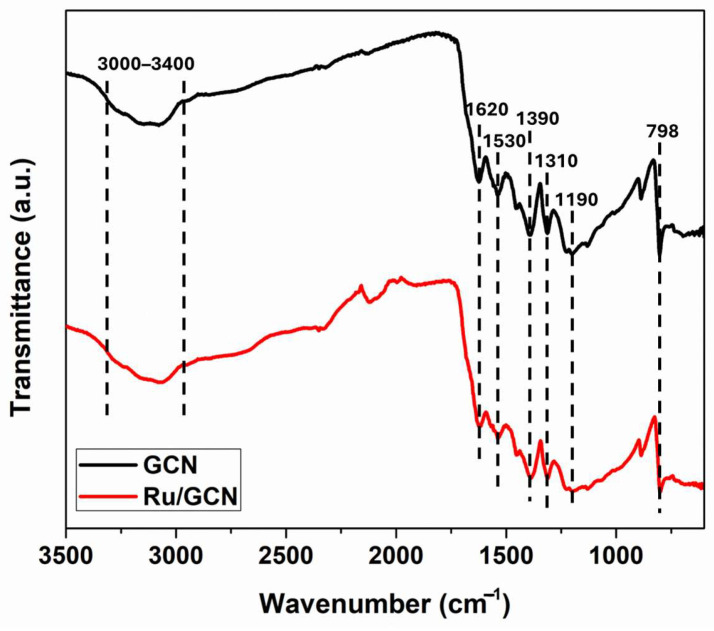
FTIR spectra of GCN and Ru/GCN.

Figure 3 presents the Fourier transform infrared (FTIR) spectra of GCN and Ru/GCN, showing many similarities between the two graphs. There is a broad absorption band between 3000 and 3400 cm^−1^ in both GCN and Ru/GCN, attributed to the N–H stretching vibrations of spherical amino groups and the O–H stretching vibrations of hydroxyl that may be located on the surface [43]. The peak at 1620 cm^−1^ can be related to the C=N stretching vibrations in GCN. The peaks in the range of 1190–1530 cm^−1^ can be ascribed to the C–N stretching vibrations of aromatic rings. The sharp peak at 798 cm^−1^ corresponds to the breathing mode of the *s*-triazine ring in GCN [44]. At the same time, it can be seen that the intensity and width of the peaks for the Ru/GCN material are slightly smaller compared to those for GCN, which can be explained by the interaction that exists between the ruthenium nanoparticles and the GCN support material.

**Figure 4:**
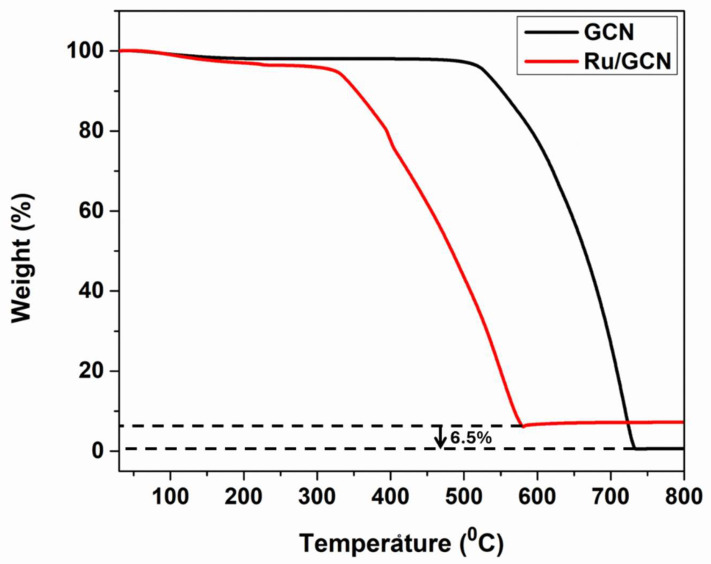
TGA profiles of GCN and Ru/GCN.

Thermogravimetric analysis (TGA) was performed in the presence of N_2_ gas to determine the thermal stability of the GCN and Ru/GCN samples (Figure 4). It can be observed that 5% weight loss occurs up to 180 °C for both GCN and Ru/GCN, corresponding to the evaporation of adsorbed water molecules from the samples’ surface. In the case of GCN, it remains stable from 180 °C up to 500 °C, meaning that there is no weight loss; then, a sharp drop in weight can be observed at temperatures around 500 °C to 700 °C, which is consistent with the fact that the aromatic network C–N of the heterocyclic ring begins to disintegrate at these temperatures. For the Ru/GCN nanocomposite, it shows stability up to 220 °C and starts decomposing continuously up to 600 °C. Moreover, the Ru/GCN sample curve reaches a plateau at a weight percentage that is 6.5% higher than that of the GCN sample, which corresponds to the percentage of metallic ruthenium nanoparticles present in the samples, as these nanoparticles do not decompose in the measured temperature range.

**Figure 5:**
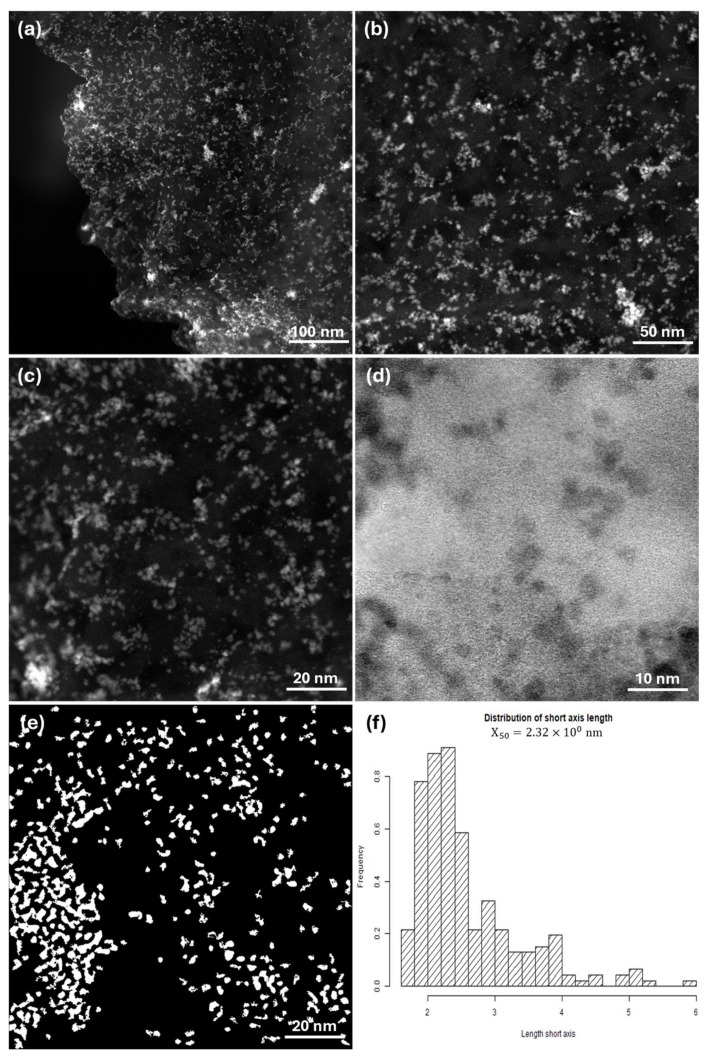
STEM images of Ru/GCN (**a**–**e**) and (**f**) particle size distribution curve of Ru nanoparticles in Ru/GCN.

The elemental composition and morphology of the synthesized Ru/GCN catalyst were characterized using scanning transmission electron microscopy (STEM). As shown in Figure 5, Ru particles are dispersed uniformly on the surface of the GCN nanosheets. In Figure 5a–c, the white areas represent Ru nanoparticles, while the dark areas correspond to GCN. The mean size of the Ru nanoparticles on the GCN surface is approximately 1–3 nm. Figure 5d displays an inverse contrast, with Ru particles appearing black against a white GCN background. Figure 5e clearly illustrates the size and distribution of the Ru particles, which are shown in white. The Ru nanoparticles are well distributed across the entire support surface. The particle distribution curve in Figure 5f indicates that the most populated particle size range is between 1.8 and 2.5 nm. In addition, energy dispersive spectroscopy (EDS) analysis further confirmed the presence of the constituent elements C, N, O, and Ru in the prepared material, with ruthenium constituting 6.43%, consistent with the value obtained from TGA analysis (Figure S1 and Table S1). Elemental mapping of the Ru/GCN catalyst (Figure S2) shows the presence and distribution of carbon, nitrogen, oxygen, and ruthenium in the material. To study the distribution of elements, SEM-EDS measurements were performed. The results include a SEM image of the Ru/GCN catalyst, a table detailing the elemental composition, and elemental mapping (Figures S3–S5, Table S2). These analyses confirmed the presence of 6.5 wt% Ru metal in the Ru/GCN catalyst.

**Figure 6:**
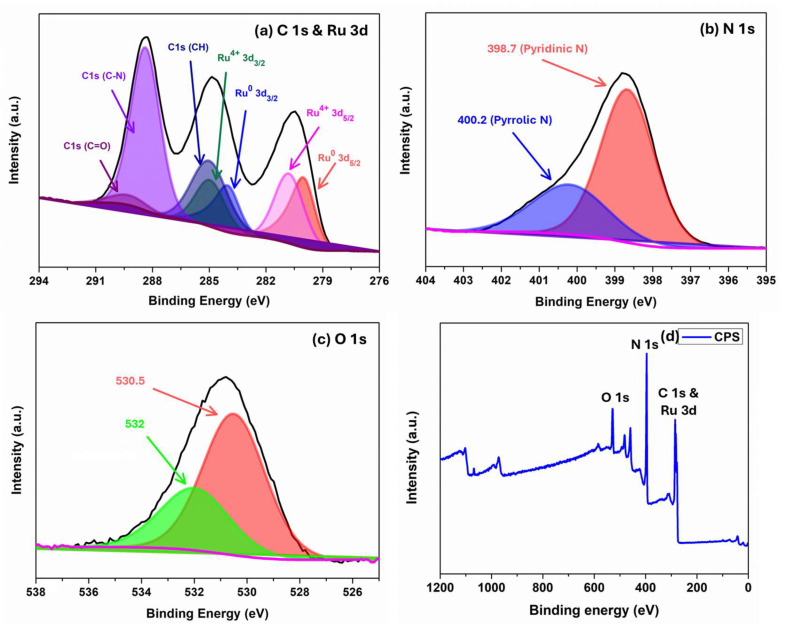
Deconvoluted XPS spectra of (**a**) C1s and Ru 3d, (**b**) N1s, and (**c**) O1s and (**d**) survey spectrum of elements in Ru/GCN catalyst.

The elemental composition and the oxidation state of each element of Ru/GCN were studied using X-ray photoelectron spectroscopy (XPS). The survey spectrum scan clearly indicates the presence of carbon, nitrogen, oxygen, and ruthenium elements (Figure 6d). The deconvoluted spectra of C1s and Ru3d, N1s, and O1s for the Ru/GCN catalyst are shown in Figure 6a–c. The spectrum of C1s shows peaks at 285.0, 288.3, and 289.3 eV. The peak at 285.0 corresponds to graphitic carbon, the peak at 288.3 corresponds to the N–C=N species, and the peak at 289.3 corresponds to the C–N–C species in the support material. The deconvoluted N1s spectrum of Ru/GCN shows peaks at 398.7 and 400.2 eV for the pyridinic nitrogen species (C=N–C) and pyrrolic nitrogen, respectively [45]. The O1s spectrum shows two peaks at 530.5 and 532.0 eV. The peak at 530.5 can be attributed to the C–OH peak corresponding to surface hydroxyls or H_2_O molecules, and the peak at 532.0 corresponds to the oxygen atom present in the small amount of RuO_2_ in the material [46,47]. The deconvoluted spectrum of Ru3d shows peaks at 280.3, 282.2, 284.5, and 286.1 eV. The peaks at 280.3 and 284.5 represent the Ru3d_5/2_ and Ru3d_3/2_ electronic state of the Ru metal in the zero-oxidation state, respectively. Similarly, the peaks at 282.2 and 286.1 represent the Ru3d_5/2_ and Ru3d_3/2_ electronic state of the Ru metal in the Ru^4+^ oxidation state, respectively [48,49].

### 3.2. Catalytic Activity

Hypophosphite is an excellent source of elemental hydrogen production (Equation (2)). Hypophosphite has two sources of hydrogen that can statistically participate in the reaction with water, and therefore it has a higher activity than other hydrogen sources.

(2) $$H_{2}{PO}_{2}^{-}+H_{2}O\to H_{2}{PO}_{3}^{-}+H_{2}$$

In this study, we investigated the optimal conditions of hydrogen release from an aqueous solution of hypophosphite using Ru/GCN as a catalyst. Ruthenium has excellent catalytic potential as it is a stable metal compared to nickel and is cheap compared to palladium, which is expensive and has limited industrial applications. Table 1 summarizes the various parameters tested to optimize the process, with all experiments conducted for 30 min. It can be seen that the higher the temperature, the higher the yield of hydrogen; at 85 °C, 100% conversion was reached within 30 min (Table 1, entries 1–3). Additionally, when the amount of catalyst was increased from 25 g to 50 g, the conversion rate also doubled, reaching 100%. However, further increasing the mass of the catalyst in the process showed a decrease in yield (Table 1, entries 3–6). Furthermore, it was found that the lower the concentration of the hypophosphite solution, the better the catalytic activity of Ru/GCN and the higher the percentage of hydrogen yield (Table 1, entries 6–9). However, for higher concentrations of hypophosphite solution, it was demonstrated that there was an improvement in the yield percentage when the amount of catalyst was also increased, though it still did not reach 100% conversion (Table 1, entries 8–11). It can be concluded that the optimal reaction conditions for this process which yield full conversion are a temperature of 85 °C, 50 g of the Ru/GCN catalyst, and a hypophosphite solution concentration of 4 M for 30 min.

**Figure 7:**
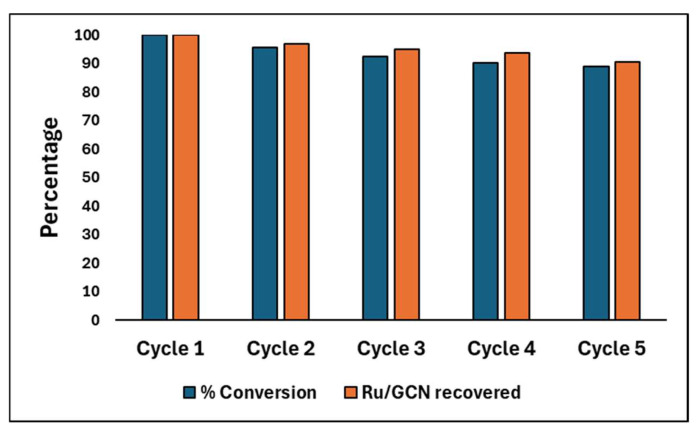
Recyclability test of Ru/GCN catalyst over five cycles.

In addition to determining the optimal catalytic conditions, the recyclability of the Ru/GCN catalyst was assessed over five cycles using the percentage of Ru/GCN recovered and the corresponding % conversion (Figure 7). In the first cycle, the catalyst exhibited 100% conversion. In the subsequent cycles, a gradual decline in both recovery and conversion was observed. By the second cycle, the recovery dropped to 97%, with a conversion rate of 95.7%. This trend continued, with the third cycle showing a recovery of 95% and a conversion rate of 92.5%. The fourth cycle resulted in a recovery of 93.6% and a conversion rate of 90.2%. By the fifth cycle, the recovery rate was 90.6%, and the conversion rate decreased to 88.9%. These results indicate that while the Ru/GCN catalyst retains significant activity over multiple cycles, there is a measurable decrease in both the amount of catalyst recovered and its catalytic efficiency, with a total decrease in conversion of 11.1% from the first to the fifth cycle.

**Figure 8:**
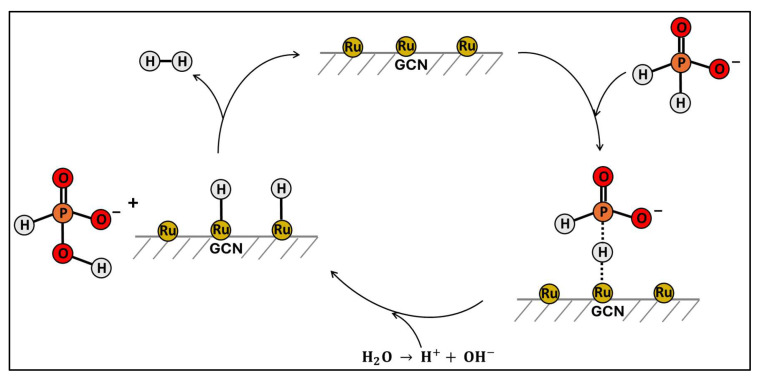
Proposed catalytic cycle for the hydrogen generation from hypophosphite catalyzed by Ru/GCN.

The proposed mechanism for hydrogen generation from the aqueous solution of hypophosphite catalyzed by Ru/GCN involves an electrochemical process with electron transfer reaction (Figure 8). The hypophosphite ion has a distorted tetrahedral structure, with a phosphorus atom at the center and two hydrogen atoms and two oxygen atoms at the vertices. During the catalytic interaction with the Ru/GCN surface, the hypophosphite ion adsorbs onto the Ru catalyst, and one of its hydrogen atoms forms a covalent bond with the Ru surface. This interaction weakens the P–H bond, leading to its cleavage. Subsequently, the hydrogen atom is replaced by a hydroxide ion from water, resulting in the transformation of hypophosphite into phosphite. The reaction produces electrons, atomic hydrogen, and protons, which combine to form molecular hydrogen. Finally, the catalyst is regenerated and returns to another catalytic cycle, ensuring continuous hydrogen production.

## 4. Conclusions

This study highlights hypophosphite as a promising source for hydrogen generation due to its commercial availability, low cost, safety, and environmental friendliness. Hypophosphite’s ability to release clean hydrogen without byproducts and its high stoichiometric potential for hydrogen production make it particularly appealing. We proposed the use of Ru/GCN as a catalyst for hydrogen production from hypophosphite, emphasizing its catalytic efficiency and the significant role of nanomaterials in enhancing catalytic performance and stability. The catalyst was characterized using various spectroscopic methods, and process conditions were optimized to achieve maximum hydrogen yield. The optimal reaction conditions for full conversion were determined to be a temperature of 85 °C, 50 g of Ru/GCN catalyst, and a hypophosphite solution concentration of 4 M for 30 min. Additionally, the recyclability test showed that the Ru/GCN catalyst maintained its catalytic activity over five cycles, with only a 12% decrease from the first to the fifth cycle. These findings demonstrate the viability of Ru/GCN nanocomposites for practical applications in sustainable hydrogen production.

## Figures and Tables

**Table 1:** The optimization of the hydrogen generation reaction from hypophosphite catalyzed by Ru/GCN.

Entry No.	Temp. (°C)	Ru/GCN (mg)	NaH_2_PO_2_ (M)	Yield (%)
1	60	50	4	27.8
2	75	50	4	66.7
3	85	50	4	100
4	85	25	4	52.1
5	85	100	4	67.7
6	85	200	4	64.6
7	85	50	2	75.0
8	85	50	6	36.8
9	85	50	8	22.4
10	85	100	6	64.6
11	85	200	8	49.0

## Data Availability

Data is contained within the article or Supplementary Materials.

## Supplementary Materials

The following supporting information can be downloaded at: https://www.mdpi.com/article/10.3390/nano14141187/s1, Figure S1: TEM image with elemental mapping and EDS data of Ru/GCN catalyst; Table S1: Elemental composition of Ru/GCN catalyst; Figure S2: Elemental mapping of Ru/GCN catalyst (from TEM analysis); Figure S3: SEM image of Ru/GCN catalyst; Table S2: Elemental composition of Ru/GCN catalyst; Figure S4: Elemental quantification of Ru/GCN catalyst; Figure S5: Elemental mapping of Ru/GCN catalyst (from SEM analysis).
