# Supplementary material for: Ru/GCN Nanocomposite as an Efficient Catalyst for Hydrogen Generation from Sodium Hypophosphite
Source: Nanomaterials (Basel). 2024 Jul 12;14(14):1187. doi: 10.3390/nano14141187 (PMC11280338; doi:10.3390/nano14141187)
Supplement: Supplementary file 1 [file nanomaterials-14-01187-s001.zip › nanomaterials-3084838-supplementary.pdf]

Supplementary Materials

Ru/GCN Nanocomposite as an Efficient Catalyst for Hydrogen Generation from Sodium Hypophosphite

Ron Shirman †, Sourav Chakraborty † and Yoel Sasson *

Casali Center of Applied Chemistry, Institute of Chemistry, The Hebrew University of Jerusalem, Jerusalem 9190401, Israel; ron.shirman@mail.huji.ac.il (R.S.); sourav.chakraborty@mail.huji.ac.il (S.C.)
* Correspondence: ysasson@huji.ac.il
† These authors contributed equally to this work.

Table of Contents

S.No.	Description	Page Number
1	Figure S1 – TEM image with elemental mapping and EDS data of Ru/GCN catalyst.	S2
1	Table S1 – Elemental composition of Ru/GCN catalyst.	S2
2	Figure S2 – Elemental mapping of Ru/GCN catalyst (from TEM analysis).	S3
3	Figure S3 – SEM image of Ru/GCN catalyst.	S4
2	Table S2 – Elemental composition of Ru/GCN catalyst.	S4
4	Figure S4 – Elemental quantification of Ru/GCN catalyst.	S4
5	Figure S5 – Elemental mapping of Ru/GCN catalyst (from SEM analysis).	S5

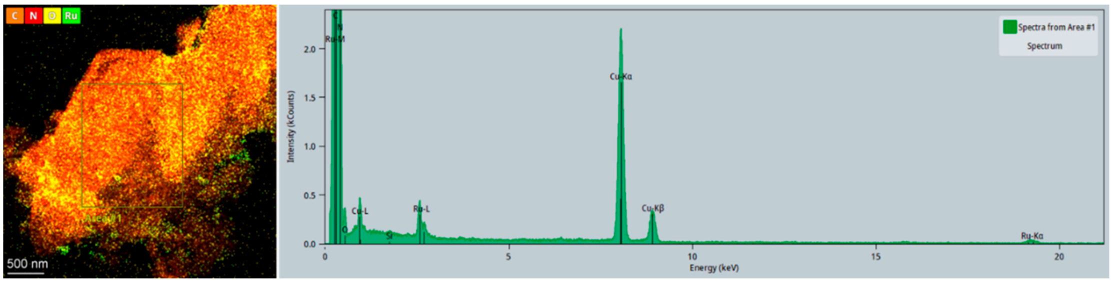

Figure S1. TEM image with elemental mapping and EDS data of Ru/GCN catalyst.

Table S1. Elemental composition of Ru/GCN catalyst.

Element	Family	Atomic fraction (%)	Atomic error (%)	Mass fraction (%)	Mass error (%)	Fit error (%)
C	K	55.11	4.81	48.41	4.35	0.34
N	K	43.65	4.92	44.72	4.96	0.49
O	K	0.38	0.11	0.44	0.13	20.18
Ru	K	0.87	0.13	6.43	0.95	0.62

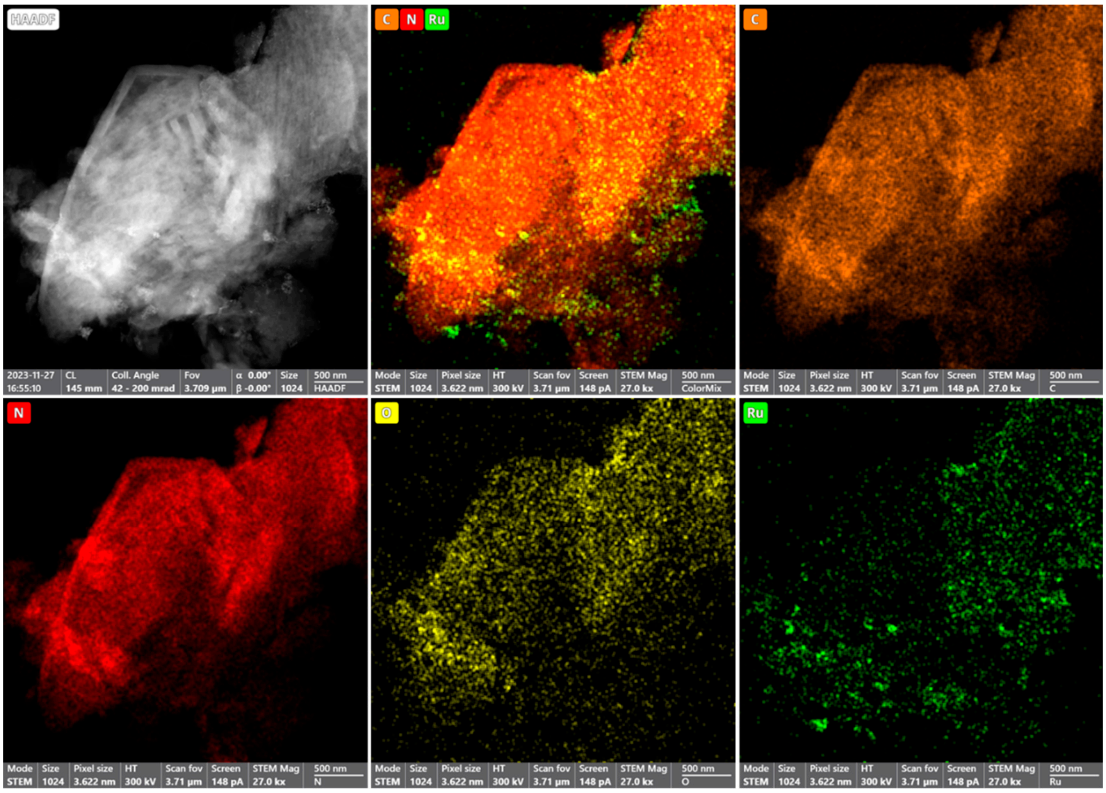

Figure S2. Elemental mapping of Ru/GCN catalyst (from TEM analysis).

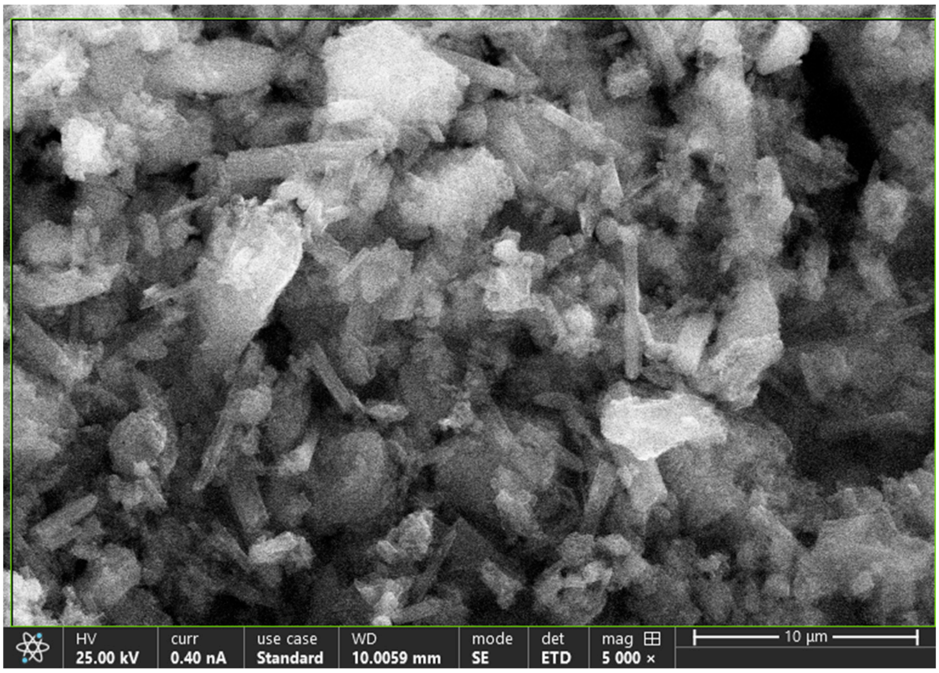

Figure S3. SEM image of Ru/GCN catalyst.

Table S2. Elemental composition of Ru/GCN catalyst.

Element	Atomic %	Atomic % Error	Weight %	Weight % Error	Net Counts
Ru	0.4	0.0	6. 5	0.0	25 873
C	25.3	0.2	33.0	0.2	112 275
N	60.2	0.5	59.7	0.5	66 285
O	14.1	0.4	0.8	0.5	9 687

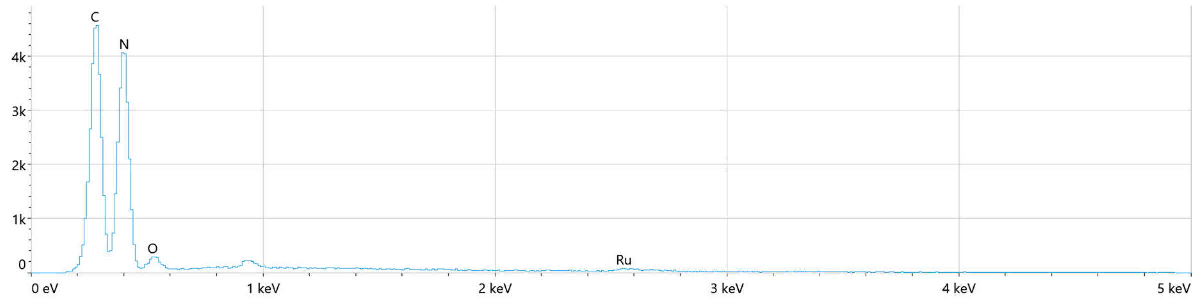

Figure S4. Elemental quantification of Ru/GCN catalyst.

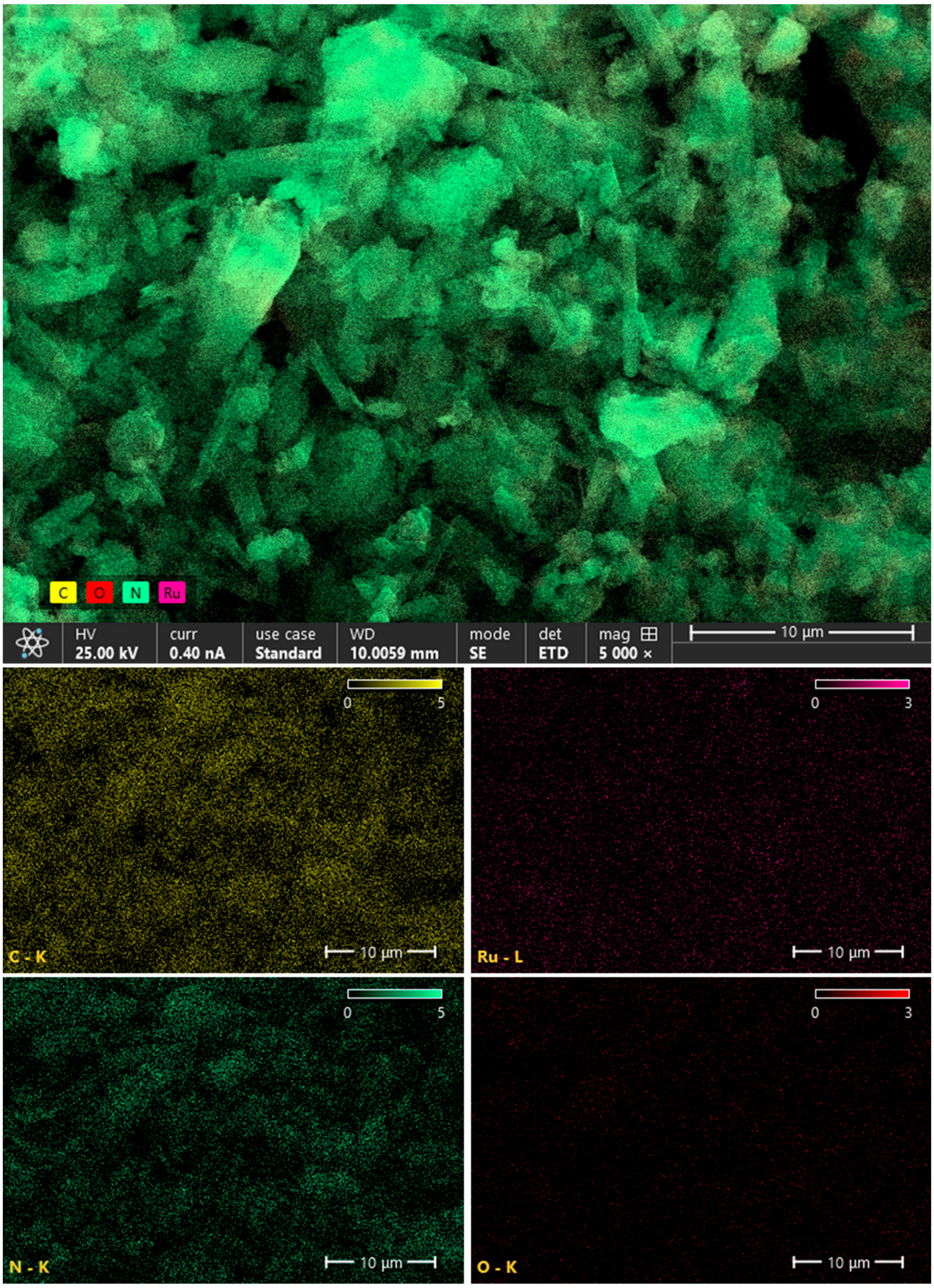

Figure S5. Elemental mapping of Ru/GCN catalyst (from SEM analysis).
